# Successful management of pembrolizumab-associated Stevens-Johnson syndrome/toxic epidermal necrolysis in a patient with uterine serous carcinoma: a case report

**DOI:** 10.3389/fimmu.2026.1872034

**Published:** 2026-07-01

**Authors:** Tasabeeh Hamad, Ana Maria Serrano, Shaher Al Kaabi, Maiya Al Bahri, Kefah Hassan, Bushra Salman, Ikram A. Burney

**Affiliations:** 1 Pharmacy Department, Sultan Qaboos Comprehensive Cancer Care and Research Center, University Medical City, Muscat, Oman; 2 Dermatology Unit, Family Medicine and Community Health (FAMCO) Department, Sultan Qaboos University Hospital, University Medical City, Muscat, Oman; 3 Dermatology Resident, Oman Medical Specialty, Muscat, Oman; 4 Department of Pathology, Sultan Qaboos University Hospital and College of Medicine and Health Sciences, University Medical City, Muscat, Oman; 5 Nursing Department, Sultan Qaboos Comprehensive Cancer Care and Research Center, University Medical City, Muscat, Oman; 6 Pharmacy Department, National Hematology and Bone Marrow Transplantation Center, University Medical City, Muscat, Oman; 7 Women Health Program, Sultan Qaboos Comprehensive Cancer Care and Research Center, University Medical City, Muscat, Oman

**Keywords:** cyclosporine, etanercept, immune checkpoint inhibitors, pembrolizumab, Stevens-Johnson syndrome, toxic epidermal necrolysis

## Abstract

Stevens-Johnson syndrome (SJS) and toxic epidermal necrolysis (TEN) are rare, life-threatening cutaneous adverse reactions associated with immune checkpoint inhibitors (ICIs). Evidence guiding optimal management remains limited. We report the case of a 75-year-old woman with stage IV endometrial carcinoma who developed extensive epidermal necrosis three weeks after the second cycle of maintenance pembrolizumab. Skin biopsy confirmed full-thickness epidermal necrosis, and the SCORTEN score was 3, predicting a high risk of mortality. There was no significant ocular or mucosal involvement. The patient was treated with etanercept, cyclosporine, and pulse methylprednisolone, followed by tapering oral corticosteroids alongside meticulous supportive care. Epidermal detachment stabilized rapidly after therapy and early re-epithelialization occurred within 10 days. The case highlights the importance of early recognition and judicious use of combination immunomodulatory therapy leading to improved outcomes. Multidisciplinary management is essential.

## Highlights

- SJS/TEN is a rare but life-threatening immune-related adverse event of pembrolizumab.
- Combination therapy with Etanercept, Cyclosporine, and corticosteroids may accelerate stabilization.
- Close monitoring of Cyclosporine levels is necessary to minimize toxicity.
- Multidisciplinary care improves clinical outcomes.

## Background

SJS and TEN are severe cutaneous reactions characterized by extensive necrosis and detachment of the epidermis and mucous membranes (1). They are rare conditions, with an estimated incidence of 5–6 cases per million annually, and a higher prevalence among females (2, 3).

Most cases are drug-induced, commonly associated with allopurinol, antiepileptics, sulphonamides, and non-steroidal anti-inflammatory drugs (NSAIDs) (4–6).

More recently, anticancer therapies, including tyrosine kinase inhibitors and immune checkpoint inhibitors (ICIs) such as ipilimumab, pembrolizumab, and nivolumab have also been implicated (7, 8).

In a subset of cases, no clear causative agent is identified, with infections such as Mycoplasma pneumoniae contributing to some cases (9, 10).

Although dermatologic immune-related adverse events (irAEs) are relatively common with ICIs, SJS/TEN remains rare but potentially life-threatening. A meta-analysis of randomized clinical trials demonstrated that patients receiving ICIs had approximately a four-fold increased risk of SJS/TEN compared with patients receiving non-ICI therapies or standard anticancer treatments. These reactions typically occur within the first few weeks of treatment, with a median onset of around 3–4 weeks and are associated with substantial mortality rates approaching 30–40% in severe cases (11, 12).

The pathogenesis of SJS/TEN is driven by immune-mediated keratinocyte apoptosis, primarily through cytotoxic T-cell activation involving Fas–Fas ligand interactions and perforin–granzyme pathways. In patients receiving ICIs, blockade of programmed cell death protein 1 (PD-1) can result in exaggerated T-cell activity and loss of tolerance to epidermal antigens. Pro-inflammatory cytokines, particularly tumor necrosis factor-alpha (TNF-α), further amplify this response, supporting the use of targeted immunomodulatory therapies (13).

Management of SJS/TEN remains largely supportive and requires early recognition, prompt withdrawal of the causative agent, and multidisciplinary care. Key measures include fluid and electrolyte replacement, wound care, infection prevention, and adequate analgesia. Systemic therapies remain an area of ongoing investigation. Corticosteroids may reduce immune-mediated damage when used early, though infection risk is a concern. Intravenous immunoglobulin (IVIG) has been used to inhibit Fas-mediated apoptosis, while cyclosporine suppresses T-cell activation and may limit disease progression. More recently, TNF-α inhibitors, particularly etanercept, have shown promise, with studies suggesting improved survival and faster re-epithelialization. However, robust comparative evidence for optimal therapy is still limited (14).

Given the rarity of ICIs–associated SJS/TEN and the lack of standardized treatment guidelines, individual case reports play an important role in guiding clinical management. We report a case of pembrolizumab-induced SJS/TEN, successfully managed with early combination immunomodulatory therapy. This case aims to highlight the clinical presentation, diagnostic challenges, and therapeutic approach to immunotherapy-induced SJS/TEN, with emphasis on the role of coordinated multidisciplinary management alongside early targeted intervention.

## Case presentation

A 75-year-old female diagnosed with stage IV high-grade Serous carcinoma of endometrium (inguinal lymph nodes involvement). On comprehensive geriatric assessment scan, G8 tool score was 13/17. The tumor showed the following molecular: estrogen receptor (ER) negative, progesterone receptor (PR) negative, mismatch repair protein proficient (pMMR), p53abn, HER-2 3 +. Next generation sequencing also showed p53 mutation, and ERBB2 amplification. The patient was treated with systemic chemotherapy (carboplatin and paclitaxel), followed by pembrolizumab maintenance treatment. Comorbid illnesses included hypertension, diabetes mellitus, osteoporosis, and diastolic dysfunction, for which she received Dapagliflozin, Amlodipine, Furosemide, and Metformin.

## Clinical course and findings

**Figure 1:**
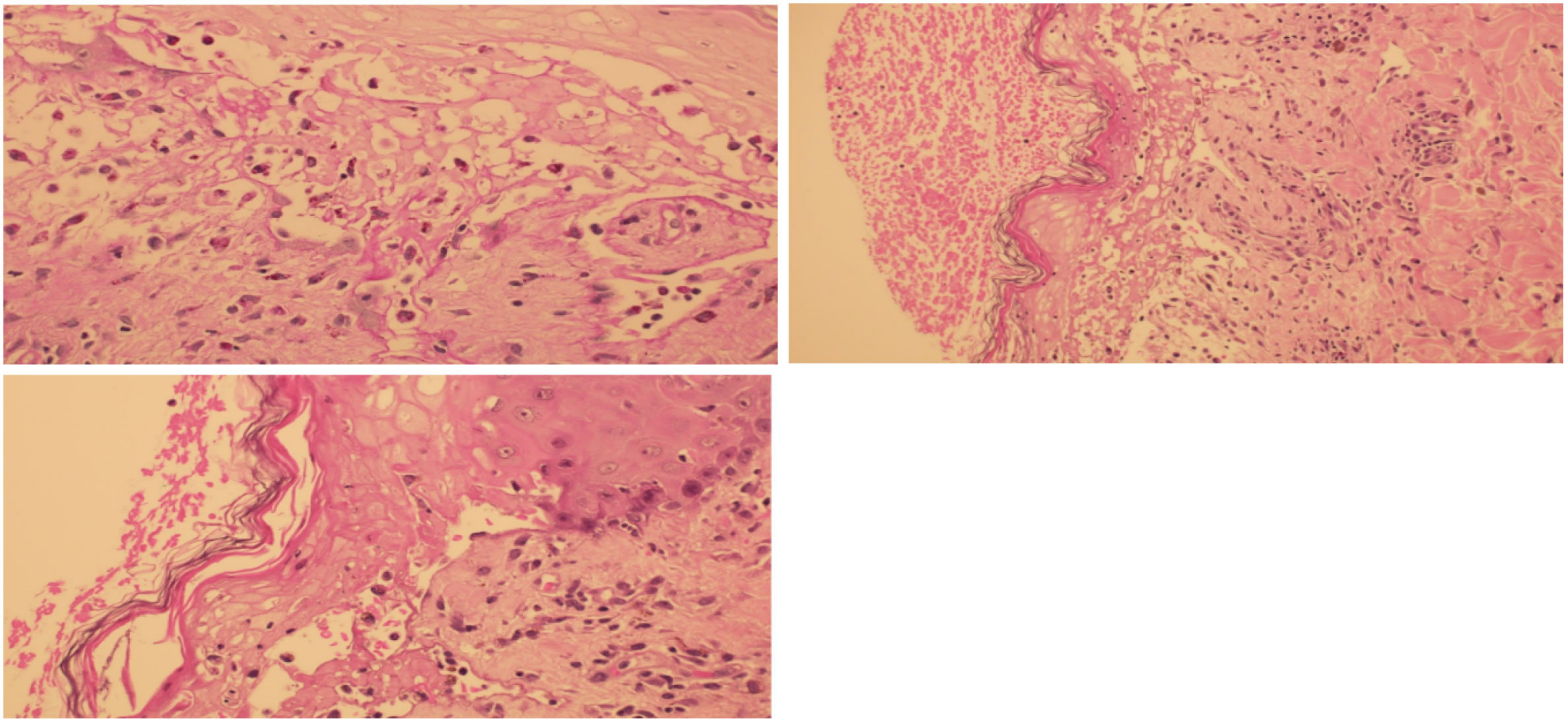
Skin punch biopsy showing extensive full-thickness epidermal keratinocyte necrosis with dermoepidermal separation, dermal edema, and mild superficial perivascular inflammatory infiltrates (H&E stain; original magnifications ×20 and ×40).

Three weeks after completion of the second cycle of maintenance pembrolizumab, the patient presented to the day-care unit for the planned third cycle, reporting severe right limb pain and shortness of breath. In view of these symptoms, the treatment with pembrolizumab was postponed, and the patient was admitted for further evaluation of breathlessness and pain control. She was started on Celecoxib, Pregabalin, and Tramadol. Five days after hospitalization, the patient developed acute-onset skin peeling with significant ulceration, predominantly affecting dependent areas, associated with severe burning pain. There was no history of fever or other acute systemic symptoms. Subsequently the patient developed generalized pruritus, and over the following days, a progressively painful and pruritic eruption developed over the back. The lesions initially appeared as small erythematous macules, which became dusky, progressing to flaccid bullae, and eventually led to epidermal detachment with increasing extent and severity involving most of the back. On physical examination, dusky, tender maculopapular eruption with areas of epidermal necrosis and skin detachment were noted predominantly over the back, along with multiple flaccid bullae. Nikolsky and Asboe–Hansen signs were positive. Mucosal examination revealed mild conjunctival erythema with minimal discharge, while the oral mucosa showed dryness only, without fissures, ulceration, or hemorrhagic crusting. There was no nasal mucosal involvement. Disease severity assessment using the SCORTEN scale (**Table 1**) yielded a score of 3, corresponding to an estimated mortality risk of 35%. A punch skin biopsy from the right upper back revealed a diagnosis consistent with SJS/TEN. There was no evidence of malignancy, as shown in **Figure 1**.

**Table 1:** SCORTEN severity assessment at presentation.

Parameter	Present in patient	Score
Age > 40 years	Yes	1
Presence of malignancy	Yes	1
Heart rate > 120	No	0
Initial epidermal detachment >10% BSA	Yes	1
Serum urea >10 mmol/L	No	0
Serum glucose >14 mmol/L	No	0
Serum bicarbonate <20 mmol/L No 0	No	0
Total **SCORTEN**		3

-SSCOTEN 3 predicted in hospital mortality approximately 35%

At the onset of skin ulceration, all potential offending medications were immediately discontinued, including Celecoxib. Given the temporal association with pembrolizumab, a diagnosis of pembrolizumab-induced SJS/TEN was made, and the drug was permanently discontinued. The patient was treated with two doses of Etanercept, Cyclosporine, and Methylprednisolone pulse therapy, followed by a transition to oral Prednisolone with a gradual taper. Cyclosporine was initiated at 5 mg/kg/day (oral equivalent), converted to equivalent intravenous dosing. The initial trough level measured prior to the third dose was (660 ng/mL). In response to this high level, the dose was reduced to 4 mg/kg/day (oral equivalent), with a repeat trough of (78 ng/mL), and continued at this dose. The patient received cyclosporine for a total of 11 days, 8 days intravenously followed by 3 days orally, with a tapering schedule from 5 to 3 mg/kg/day before discontinuation. Supportive management included gentle handling of the skin, saline cleansing, application of topical Fusidic acid, and use of non-adherent dressings to promote healing and prevent secondary infection. Throughout the course of treatment, close monitoring of renal function, blood pressure, blood glucose levels, serum electrolytes, and Cyclosporine trough levels was maintained.

Following initiation of immunomodulatory therapy, there was a gradual clinical improvement. Progression of epidermal detachment stabilized, and areas of epidermal loss began to show early signs of re-epithelialization. Following the second dose of Etanercept, cutaneous lesions stabilized, and no further new bullae developed. During follow-up, there was no evidence of oral, nasal, or significant ocular mucosal involvement. The previously noted conjunctival erythema did not progress, and no long-term ocular complications were observed during hospitalization. The patient remained hemodynamically stable, with renal function, electrolytes, blood glucose, blood pressure, and Cyclosporine levels maintained within the therapeutic range under close monitoring. Ten days after the first eruption of bullae, the patient showed continued improvement in skin integrity with reduction in pain and tenderness. The patient was discharged to home and followed up in the clinic. Two months after the episode, there were no pruritic or fresh bullae, and the macular rash had disappeared. The timeline of the clinical episode is shown in **Table 2**.

**Table 2:** Timeline of clinical episode.

Time point	Clinical events and diagnostic findings	Therapeutic interventions
3 Weeks post-Cycle 2	Patient presented with severe right limb pain and shortness of breath.	Pembrolizumab Cycle 3 postponed; Admitted for evaluation.
Day 1–4 of Admission	Evaluation of pain and breathlessness.	Started on Celecoxib, Pregabalin, and Tramadol.
Day 5 of Admission	Acute-onset skin peeling, significant ulceration (dependent areas), severe burning pain. No fever.	Discontinued all potential offending drugs (including Celecoxib).
Day 6–8 of Admission	Generalized pruritus; eruption of dusky erythematous macules progressing to flaccid bullae on the back.	Diagnosis: Pembrolizumab-induced SJS/TEN. Permanent discontinuation of pembrolizumab.
Day 9 of Admission	Physical Exam: Epidermal necrosis/detachment over the back. Positive Nikolsky and Asboe–Hansen signs. Mild conjunctival erythema.	Initiated Methylprednisolone pulse therapy, Etanercept (Dose 1), and Cyclosporine (5 mg/kg/day).
Day 11 of Admission	Cyclosporine trough level: 660 ng/mL (High).	Cyclosporine dose reduced to 4 mg/kg/day.
Day 12–14 of Admission	Clinical stabilization; repeat Cyclosporine trough: 78 ng/mL. No new bullae.	Etanercept (Dose 2) administered. Continued supportive care (Fusidic acid, non-adherent dressings).
Day 17–20 of Admission	Signs of early re-epithelialization. Stabilization of cutaneous lesions.	Transitioned from IV to Oral Cyclosporine (3-day taper) and Oral Prednisolone (gradual taper).
Post-Discharge/Follow-up	Complete stabilization. No oral, nasal, or significant ocular mucosal sequelae.	Continued Prednisolone taper; pembrolizumab permanently discontinued.

## Discussion

We present the case of pembrolizumab-induced SJS/TEN. The adverse reaction was severe, and the risk of mortality was 35%. The patient was managed by a multidisciplinary team consisting of medical Oncologist, Dermatologist, clinical pharmacist and the specialized nursing services. Epidermal detachment stabilized within the first three days of treatment, and re-epithelialization was evident by the end of the week. The patient was discharged home in two weeks, and remained free of any further epidermal necrolysis at 2-month follow up. The patient had been exposed to at least three agents previously reported to cause SJS/TEN. Pregabalin has been associated with isolated delayed hypersensitivity reactions, ranging from mild maculopapular rashes to severe cutaneous adverse reactions (SCARs) such as DRESS, SJS, and TEN, SCARs are rare adverse reactions. In the FDA Adverse Event Reporting System (FAERS) data, 4 cases of SJS/TEN were identified among 19,029 Pregabalin adverse event reports (~0.02%). The background incidence of SJS/TEN in the general population is approximately 1.5–8.3 cases per 1,000,000 person-years (15). These reactions are immunologically mediated, typically non–dose-related and involve T-cell–mediated type IV hypersensitivity. Onset is variable, often occurring days to weeks after exposure, but may be more rapid upon re-exposure (16, 17). Celecoxib, a selective COX-2 inhibitor, has been linked to both immediate (IgE-mediated) and delayed hypersensitivity reactions (18). SCARs, including SJS and TEN, have been reported but are exceedingly rare. Post-marketing FAERS data estimate an SJS/TEN reporting rate of approximately 6 cases per 1,000,000 person-years, markedly lower than that of high-risk drugs such as antiepileptics or sulphonamides (19). Delayed reactions range from mild maculopapular eruptions and fixed drug eruptions to severe SCARs, including SJS/TEN (20), typically arising 1–8 weeks after treatment initiation. Although the patient had received Celecoxib for only 6 days, a delayed hypersensitivity reaction cannot be excluded. The diagnosis of pembrolizumab-induced SJS was supported by the ALDEN (Algorithm of Drug Causality for Epidermal Necrolysis) assessment (21). Pembrolizumab achieved an ALDEN score of 6, corresponding to probable causality (**Table 3**). This assessment was based on the compatible temporal relationship between drug exposure and symptom onset, the clinical course following drug withdrawal, the documented association of pembrolizumab with SJS/TEN, and the absence of a more likely alternative explanation. Although the patient had also been exposed to pregabalin and celecoxib, their shorter duration of exposure and substantially lower reported association with SJS/TEN made them less likely causative agents. Collectively, these findings support pembrolizumab as the most probable trigger of the patient’s SJS/TEN.

**Table 3:** ALDEN (Algorithm of drug causality for epidermal necrolysis) assessment of pembrolizumab as the suspected causative agent.

ALDEN criterion	Assessment for pembrolizumab	Score
Delay from initial drug exposure to onset of reaction	Compatible/suggestive (reaction occurred after 2 cycles; approximately 6–8 weeks after initiation)	+3
Drug present in body at index day	Present	0
Pre-challenge/Rechallenge	Not applicable	0
Dechallenge (drug withdrawal)	Improvement after withdrawal and treatment	+1
Notoriety of the drug (published association with SJS/TEN)	Strongly associated; several published case reports	+2
Presence of alternative causes	Possible alternative drugs (Celecoxib, Pregabalin), but less likely based on timing and known risk	0
**Total ALDEN Score**		**6**

-ALDEN 6, The suspected drug has very probable causal relationship with SJS/TEN

ICIs-associated SJS/TEN remains a rare irAEs but is increasingly recognized. Cutaneous irAEs occur in up to 25% patients receiving ICIs; grade 3 or more severe irAEs occur in less than 2% (21). Life-threatening irAEs such as SJS/TEN are exceedingly rare (22, 23). The mechanism is non–dose-related, and likely immunologically mediated, involving PD-1 blockade induced T-cell hyperactivation, leading to blockade of shared antigens expressed on tumor cells and the dermo-epidermal junction, resulting in keratinocyte apoptosis. Reported onset of immune-related dermatologic toxicity typically occurs after 1–3 treatment cycles, consistent with this patient’s presentation following two cycles. Apoptosis occurs via the Fas-FasL pathway or perforin/granzyme pathway (24). PD-1 blockade leading to T-cell hyperactivation leads to a TNF-α-amplified inflammatory cascade, which provides rationale for the use of TNF-α inhibition in the management.An important consideration in this case is the classification of the epidermal necrolysis. Based on the extent of epidermal detachment involving more than 20% of BSA, together with the clinical and histopathologic findings, this case is most appropriately classified as SJS/TEN overlap syndrome. Recent literature has suggested ICIs-related epidermal necrolysis (ICIREN) may represent a distinct clinicopathologic entity from classic drug-induced SJS/TEN. Compared with conventional SJS/TEN, ICIREN may demonstrate a more indolent onset, limited mucosal involvement, prominent cutaneous disease, and prolonged inflammatory activity driven by persistent immune activation. In our patient, extensive epidermal necrolysis occurred in the setting of only mild conjunctival erythema and absence of significant oral or nasal mucosal involvement, findings that have been described in ICIREN. Nevertheless, the substantial body surface area involvement and histopathologic features remain within the spectrum of SJS/TEN overlap syndrome. Recognition of the evolving concept of ICIREN is important because it may refine the interpretation of severe irAEs and influence future therapeutic approaches (25). Although no pharmacologic treatment is definitively established for SJS, meta-analyses suggest potential benefit from anti-TNF-α antibody, Etanercept, Cyclosporine, systemic corticosteroids, or combined IVIG with corticosteroids (26). Management of SJS/TEN in this patient included combination therapy with Etanercept, Cyclosporine, and intravenous Methylprednisolone. In a multicenter observational study patients who received combination therapy with Etanercept, and corticosteroids had lower actual mortality than those with corticosteroid monotherapy and those with IVIG combined with corticosteroids (25). Furthermore, Etanercept combined with corticosteroids showed a reduced skin healing time, compared with corticosteroid monotherapy, and IVIG combined with corticosteroids therapy (24). IVIG was not utilized in this case due to its inconsistent efficacy across studies, high cost, and limited evidence of superiority over other immunomodulatory therapies.

A general limitation of this case is that the use of immunomodulatory therapies, including Etanercept and Cyclosporine, was guided by clinical response rather than objective biomarkers; serum TNF-α, interleukin, or other cytokine levels were not measured; therefore, the proposed immunologic mechanisms and the contribution of cytokine modulation to the observed clinical improvement could not be objectively evaluated.

## Conclusion

This case is one of the few documented cases of pembrolizumab-associated Stevens-Johnson syndrome/toxic epidermal necrolysis (SJS/TEN) overlap syndrome, with epidermal detachment involving more than 20% of body surface area and an estimated mortality risk of 35% based on SCORTEN. This patient was successfully managed with early recognition, and prompt initiation of combined immunomodulatory therapy consisting of Etanercept, Cyclosporine and high-dose systemic corticosteroids, together with meticulous supportive skin care. Furthermore, the outcome demonstrates the importance of a multidisciplinary team in the management of ICIs–related severe cutaneous adverse reactions.

## Data Availability

The original contributions presented in the study are included in the article/supplementary material. Further inquiries can be directed to the corresponding author.

## References

[1] Roujeau JC Stern RS . Severe adverse cutaneous reactions to drugs. N Engl J Med. (1994) 331:1272–85. doi: 10.1056/NEJM199411103311906 7794310

[2] Hsu DY Brieva J Silverberg NB Silverberg JI . Morbidity and mortality of Stevens-Johnson syndrome and toxic epidermal necrolysis in United States adults. J Invest Dermatol. (2016) 136:1387–97. doi: 10.1016/j.jid.2016.03.023 27039263

[3] Sekula P Dunant A Mockenhaupt M Naldi L Bouwes Bavinck JN Halevy S . Comprehensive survival analysis of a cohort of patients with Stevens-Johnson syndrome and toxic epidermal necrolysis. J Invest Dermatol. (2013) 133:1197–204. doi: 10.1038/jid.2012.510 23389396

[4] Mockenhaupt M Viboud C Dunant A Naldi L Halevy S Bouwes Bavinck JN . Stevens-Johnson syndrome and toxic epidermal necrolysis: assessment of medication risks with emphasis on recently marketed drugs. The EuroSCAR-study. J Invest Dermatol. (2008) 128:35–44. doi: 10.1038/sj.jid.5701033 17805350

[5] Roujeau JC Kelly JP Naldi L Rzany B Stern RS Anderson T . Medication use and the risk of Stevens-Johnson syndrome or toxic epidermal necrolysis. N Engl J Med. (1995) 333:1600–7. doi: 10.1056/NEJM199512143332404 7477195

[6] Wang YH Chen CB Tassaneeyakul W Saito Y Aihara M Choon SE . The medication risk of Stevens-Johnson syndrome and toxic epidermal necrolysis in Asians: the major drug causality and comparison with the US FDA label. Clin Pharmacol Ther. (2019) 105:112–20. doi: 10.1002/cpt.1071 29569740

[7] Saw S Lee HY Ng QS . Pembrolizumab-induced Stevens-Johnson syndrome in non-melanoma patients. Eur J Cancer. (2017) 81:237–9. doi: 10.1016/j.ejca.2017.03.026 28438440

[8] Maloney NJ Ravi V Cheng K Bach DQ Worswick S . Stevens-Johnson syndrome and toxic epidermal necrolysis-like reactions to checkpoint inhibitors: a systematic review. Int J Dermatol. (2020) 59:e183–8. doi: 10.1111/ijd.14811 32052409

[9] Chaby G Ingen-Housz-Oro S De Prost N Wolkenstein P Chosidow O Fardet L . Idiopathic Stevens-Johnson syndrome and toxic epidermal necrolysis: prevalence and patients' characteristics. J Am Acad Dermatol. (2019) 80:1453–5. doi: 10.1016/j.jaad.2018.10.058 30395917

[10] Liew YCC Choo KJL Oh CC Pang SM Yeo YW Lee HY . Mycoplasma-induced Stevens-Johnson syndrome/toxic epidermal necrolysis: case-control analysis of a cohort managed in a specialized center. J Am Acad Dermatol. (2022) 86:811–7. doi: 10.1016/j.jaad.2021.04.066 33915240

[11] Nassif A Moslehi H Le Gouvello S Bagot M Lyonnet L Michel L . Evaluation of the potential role of cytokines in toxic epidermal necrolysis. J Invest Dermatol. (2004) 123:850–5. doi: 10.1111/j.0022-202X.2004.23439.x 15482470

[12] Zhu J Chen G He Z Zheng Y Gao S Li J . Stevens-Johnson syndrome/toxic epidermal necrolysis in patients treated with immune checkpoint inhibitors: a safety analysis of clinical trials and FDA pharmacovigilance database. EClinicalMedicine. (2021) 37:100951. doi: 10.1016/j.eclinm.2021.100951 34386743 PMC8343267

[13] Saeed HN Chodosh J . Immunologic mediators in Stevens-Johnson syndrome and toxic epidermal necrolysis. Semin Ophthalmol. (2016) 31:85–90. doi: 10.3109/08820538.2015.1115255 26959133

[14] Zhou J Wang CP Li J Zhang HL He CX . Stevens-Johnson syndrome and toxic epidermal necrolysis associated with immune checkpoint inhibitors: a systematic review. Front Immunol. (2024) 15:1414136. doi: 10.3389/fimmu.2024.1414136 39072330 PMC11272453

[15] Borrelli EP Lee EY Descoteaux AM Kogut SJ Caffrey AR . Stevens-Johnson syndrome and toxic epidermal necrolysis with antiepileptic drugs: an analysis of the US Food and Drug Administration Adverse Event Reporting System. Epilepsia. (2018) 59:2318–24. doi: 10.1111/epi.14591 30395352 PMC6420776

[16] Frey N Bodmer M Bircher A Rüegg S Jick SS Meier CR . The risk of Stevens-Johnson syndrome and toxic epidermal necrolysis in new users of antiepileptic drugs. Epilepsia. (2017) 58:2178–85. doi: 10.1111/epi.13925 29027197

[17] Inoue A Sawada Y Ohmori S Omoto D Haruyama S Yoshioka M . Maculopapular type drug eruption caused by pregabalin: a case and literature review. Allergol Int. (2016) 65:351–2. doi: 10.1016/j.alit.2016.02.006 26976337

[18] La Grenade L Lee L Weaver J Bonnel R Karwoski C Governale L . Comparison of reporting of Stevens-Johnson syndrome and toxic epidermal necrolysis in association with selective COX-2 inhibitors. Drug Saf. (2005) 28:917–24. doi: 10.2165/00002018-200528100-00008 16180941

[19] Alonso JC Ortega JD Gonzalo MJ . Cutaneous reaction to oral celecoxib with positive patch test. Contact Dermatitis. (2004) 50:48–9. doi: 10.1111/j.0105-1873.2004.00271i.x 15059110

[20] Berger P Dwyer D Corallo CE . Toxic epidermal necrolysis after celecoxib therapy. Pharmacotherapy. (2002) 22:1193–5. doi: 10.1592/phco.22.13.1193.33513 12222558

[21] Sassolas B Haddad C Mockenhaupt M Dunant A Liss Y Bork K . ALDEN, an algorithm for assessment of drug causality in Stevens-Johnson syndrome and toxic epidermal necrolysis: comparison with case-control analysis. Clin Pharmacol Ther. (2010) 88:60–8. doi: 10.1038/clpt.2009.252 20375998

[22] Belum VR Benhuri B Postow MA Hellmann MD Lesokhin AM Segal NH . Characterisation and management of dermatologic adverse events to agents targeting the PD-1 receptor. Eur J Cancer. (2016) 60:12–25. doi: 10.1016/j.ejca.2016.02.010 27043866 PMC4998047

[23] Cai ZR Lecours J Adam JP Marcil I Blais N Dallaire M . Toxic epidermal necrolysis associated with pembrolizumab. J Oncol Pharm Pract. (2020) 26:1259–65. doi: 10.1177/1078155219890659 31810421

[24] Hasegawa A Abe R . Recent advances in managing and understanding Stevens-Johnson syndrome and toxic epidermal necrolysis. F1000Res. (2020) 9:F1000 Faculty Rev–612. doi: 10.12688/f1000research.24748.1 32595945 PMC7308994

[25] Brent AA Lowe L Plotzke J . Immune checkpoint inhibitor–related epidermal necrosis: terminology, pathology, and clinical implications. Arch Pathol Lab Med. (2025). doi: 10.5858/arpa.2025-0294-RA 41285158

[26] Zhang J Lu CW Chen CB Wang CW Chen WT Cheng B . Evaluation of combination therapy with etanercept and systemic corticosteroids for Stevens–Johnson syndrome and toxic epidermal necrolysis: a multicenter observational study. J Allergy Clin Immunol Pract. (2022) 10:1295–1304.e6. doi: 10.1016/j.jaip.2022.01.038 35131514

